# Social Capital as a Mediator and Moderator in the Association between Loneliness and Health, Israel as a Case Study

**DOI:** 10.3390/ijerph19063698

**Published:** 2022-03-20

**Authors:** Orna Baron-Epel, Roni Elran-Barak, Milka Donchin

**Affiliations:** 1 School of Public Health, University of Haifa, Haifa 3498838, Israel; roniebarak@gmail.com; 2 The Braun School of Public Health & Community Medicine, The Hebrew University-Hadassah, Jerusalem 9112102, Israel; milka@hadassah.org.il; 3 Israel Healthy Cities Network, Jerusalem 91072, Israel

**Keywords:** loneliness, self-rated health, psychosomatic symptoms, social capital, structural, cognitive, Arabs, Jews

## Abstract

Loneliness has been associated with poor health. Social capital (SC) could possibly prevent the ill effects of loneliness. The study aims to assess the association of loneliness with physical and mental health in four different communities in Israel and study the impact of structural and cognitive SC on that association. A cross-sectional face-to-face survey with 4620 adults in four towns was conducted. The questionnaire included self-rated health (SRH), mental health (MH), loneliness, cognitive and structural SC and socioeconomic characteristics. Logistic regression analysis and mediation and moderation effects were calculated. Loneliness was associated with worse SRH (OR = 0.4–0.5) and worse MH (OR = 2.0–10). Both SC variables were associated with health. However, towns differ in these associations. Structural SC serves as a significant mediator between loneliness and SRH in all towns and is a mediator between loneliness and MH in two towns. Cognitive social capital was a moderator between loneliness and MH in two towns. This study suggests that increasing SC could possibly compensate for loneliness and buffer its effect on health. The study reinforces the need for the performance of separate health profiles to assess possible interventions for each community, as not always can we generalize these results to all communities.

## 1. Introduction

During the last decade, loneliness has emerged as an important determinant of health [1,2,3,4]. Studies have suggested that loneliness is a major risk factor for morbidity and mortality [5,6]. It seems that loneliness is indirectly associated with these outcomes through psychological and behavioral factors [6]. During the COVID-19 pandemic, loneliness was a major concern for public health, increasing the need to understand the factors associated with it [7].

Most of the research regarding loneliness has been conducted in the developed world and individualistic societies, however, there are studies looking at other societies [8]. In a study comparing loneliness across cultures in 237 countries, findings showed that in societies that were more individualistic there were higher rates of loneliness that decreased with age [9].

In a recent editorial, a list of risk factors and negative effects associated with loneliness in old age was given. The authors suggested that building a compassionate social community could be a key approach to dealing with loneliness [10].

Social capital (SC) may help build such communities. Identifying key elements within SC that may inhibit or promote the ill effects of loneliness would be helpful for future intervention.

### 1.1. Social Capital and Health

Putnam’s SC is an umbrella term that draws together relationships within networks, norms of reciprocity, mutual support and trustworthiness [11,12]. Social capital is often defined as the norms, networks and associations that facilitate co-operative action [13]. Social capital can be divided into both structural dimensions (the number of social relationships) and cognitive dimensions (perceptions of the quality of social relationships) [14]. The current study measures cognitive SC represented by trust and reciprocity and structural SC represented by social involvement [15].

Social capital is associated with many health outcomes [16,17,18,19,20,21]. In a systematic review, Coll-Planas et al. found mixed effects of SC interventions targeting older people’s quality of life, wellbeing and self-perceived health [22]. Social capital is now considered a social determinant of the health discourse [23].

Social capital may serve as a protective factor for health and a resource for individuals and communities to improve quality of life [12,19]. However, how this works and if this is generalizable between communities is not clear [24]. The association between SC and health may be context-dependent, depending on the specific community studied [15,25].

In a previous study in Israel, Jews reported higher levels of SC compared to Arabs, except for higher rates of social contacts, and all measures of SC were associated with self-rated health among Jews. However, among Arabs, the association between SC measures and health was not significant, except for social support. Therefore, studying these two populations may have an added value for understanding the association between social attributes and health [25].

### 1.2. Social Capital and Loneliness

Loneliness and SC both seem to affect health, moreover, SC may serve as a resource by which individuals can decrease loneliness or prevent its ill effects. Studies suggest that there is an association between living in an area with high SC and lower levels of loneliness [26,27,28]. This association may not be causal, and there may be an interaction between socioeconomic status (SES) and age. A study in a suburb of Barcelona, Spain, suggested that there was an interaction between SES, neighborhood SC, age and loneliness. The authors suggest that increasing neighborhood SC could be an effective way of reducing the prevalence of loneliness [26].

Evidence is beginning to come out that promoting SC to tackle loneliness and its health effects could be an interesting approach to decrease inequalities [29]. However, not many studies have looked at all three variables simultaneously. Therefore, there is a need for more information regarding the associations between these social factors to be able to estimate the generalizability to all communities.

Over two decades of research with more than 850 studies looking at SC and health have resulted in elusive and contradictory results, especially regarding interventions [30]. Shiell et al. suggest interventions utilizing SC to improve health are especially context-specific.

Israel can serve as an interesting setting for studying different communities as it is a multicultural society with two major ethno-cultural groups: Jews and Arabs [31,32]. About one fifth of Israel’s population comprises Arabs; of them, about 85% are Muslims and 15% are mainly Christians and Druze. Jews and Arabs in Israel differ in socioeconomic status, religion, culture, and language. The Arab society is a more collectivist society in transition to a more western lifestyle. Health indicators in Israel suggest poorer health among Arabs compared to Jews [31,32]. For this study, we chose four towns that differed in ethnicity (one Jewish town, two Arab towns and one mixed town).

### 1.3. Aim of the Study

**Figure 1:**
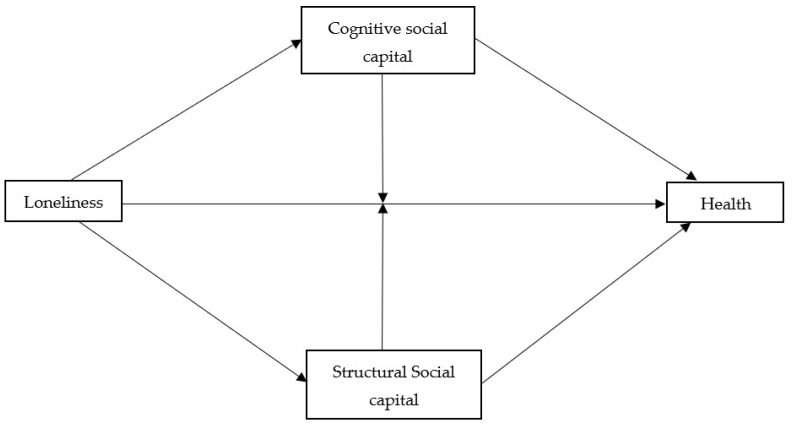
Hypothesised model depicting the association between loneliness and health.

The study’s aim was to assess the association between loneliness, cognitive and structural SC and health (mental and general self-rated health (SRH)). Additionally, we want to see whether these associations are similar in the different towns. We hypothesize that the association between loneliness and health is mediated or moderated by both cognitive and structural SC (Figure 1).

## 2. Material and Methods

### 2.1. Design and Sample

This study is a secondary analysis of data collected in cross-sectional population surveys in four towns, as part of their health profile and needs assessment. This is performed as one of their commitments to the Healthy City Network [33]. As the questionnaire is a uniform questionnaire, it allows for analyzing and comparing data from the four towns.

Four surveys were conducted in four Israeli towns during the years 2011–2016. The study population was randomly selected, in each town, as a stratified sample of households by residential area, age, and sex, by the Israel Central Bureau of Statistics (ICBS), which also provided a Kish grid for a random selection of the interviewees out of a list of inhabitants aged 22 and above, in each household. The sample size calculation in each town relied upon the following: 50% prevalence of a variable, a 95% confidence interval, a 5% relative sampling error, and 70% compliance. Interviews were conducted in person, using a structured questionnaire, at participants’ residential locations, by trained interviewers. Participation rates were 54% in the mixed Arab-Jewish town (town C), 79.3% in town A, and 89% in both towns D and B.

The sample included 1898 Arabs and 2722 Jews, altogether being 4620. Participants were residents of two Arab towns (n = 981 and 673), one Jewish town (n = 1935), and one mixed town (n = 244 Arabs, 787 Jews). The towns in the study represent two lower socioeconomic status (SES) Arab towns, level 2 and 3 out of 10, (town A and B), a mixed town with Arabs and Jews (town C, level 4 out of 10) and a Jewish town (town D, level 7 out of 10) [34]. Interviews were conducted in Arabic or Hebrew according to the town and preference of the individual.

### 2.2. Measures

Sociodemographic variables were collected in person via self-report: gender, age and education. In town C, respondents were asked to define themselves as Jews or Arabs. Religiosity was assessed using two different versions of the question, one for Arabs and one for Jews, as the terms used in Hebrew and Arabic are different. The variable was dichotomized as not religious [2] and religious [1]. For Arabs, not religious included answers 3-not so religious and 4-not religious; religious included 1-very religious and 2-religious. For Jews, not religious included 4-not so religious, and 5-not religious or secular, and religious included 1-ultra-orthodox, 2-religious and 3-traditional. 

Two health measures were analyzed: A. self-rated health (SRH) was measured using the question “*How do you evaluate your general health status?*” (1) Very good, (2) good, (3) not so good, or (4) not good. The answers were dichotomized into very good and good (good) and coded as a 1, while, not so good and not good (poor) were coded as a 0. B. Mental health was measured by asking the following question: “*During the last year have you suffered from the following problems: (1) irritability/stress/anxiety (2) sleep problems (3) sadness/depression and (4) tiredness*’’. Respondents could answer yes or no to each problem. The number of problems mentioned were summed to give a scale from 0 to 4 and dichotomized. Low mental health was defined as 0 and 1, and high-2–4. These four items were taken from the national yearly social survey of the Israeli Central Bureau of Statistics [35].

Loneliness was assessed using one item, “*Are there situations in which you feel lonely?*” [36,37], with four response options ranging from (1) never to (4) often. This was dichotomized to never or infrequently = not lonely and feeling lonely often or sometimes = lonely.

The cognitive measure of SC (trust and reciprocity) was measured asking A. “*Generally do you think 1. people can be trusted or 2. you have to be careful of trusting people*”.

B. “*Do you think that people in your town or neighborhood: 1. usually help each other, 2. usually only look after themselves*”. A composite variable was defined by adding the number of positive answers (0, 1, 2), which was further collapsed for the logistic regression as low (0) and high (1 = 1 + 2). 

Structural SC was measured by asking about participation in eight social activities. Respondents were given a list of activities and asked if they had participated in each activity during the last year, not at all (1), infrequently (2) frequently (3). The answers were added up to give a scale from 1 to 24. The activities included: sports events, meeting friends, going to the mosque or synagogue, going to parties or performances, going on hikes or tours with others, attending political or professional activities, volunteering, meeting family that do not live with the respondent.

### 2.3. Data Analysis

Data were analyzed using SPSS-25. Chi^2^ and one-way ANOVA were conducted to examine differences among the four towns. Logistic regression models were run with cognitive SC, SRH, and mental health dichotomized as the dependent variables. For the two health measures, the first step, gender, age, education, religiosity and loneliness were added to the regression. For the second step, structural or cognitive SC was added (model 1) and in the third step, an interaction between loneliness and structural or cognitive SC were added in order to test the moderation effect of SC on the association between loneliness and health (model 2). See Figure 1. This moderation effect was further elaborated by running separate regression for people with low and high SC. Linear regressions were run for structural SC as the dependent variable, with the above mentioned independent variables. A regression model was run separately for each town after finding significant interactions between town and loneliness.

The mediating models were calculated with the R mediation package, version 4.0.2, the model was performed with the library mediation. All variables were standardized. The models were first calculated while controlling for gender (1-male, 0-female), age and education, then the other variables were added. The Average Causal Mediation Effect (ACME) was the measure of the mediation effect [38].

### 2.4. Ethics

Exemption from ethical approval was granted by the University Hospital’s Ethical Committee.

The interviewees were told they were free to stop the interview at any time with no implications to them. As the interviewees agreed to answer the questionnaire this suggests informed consent.

## 3. Results

Altogether, 4620 adults were interviewed in four towns, two Arab towns (A and B), one mixed town (C) with 804 Jews and 246 Arabs in the sample, and one Jewish town (D). 

The samples from the towns differ significantly in all their characteristics except for the percent of men and women interviewed (Table 1).

The two health measures were correlated (r = 0.38 Pearson correlation coefficient). Better self-rated health (SRH) was reported by Arabs in the two Arab towns (A and B) and Jews in town C compared to Jews in town D. Arabs living in Arab towns (A and B) and Arabs in town C report better mental health than the Jews in towns C and D. In town C, the difference between Jews and Arabs for all measures was significant, except for mental health, where there was no significant difference between the two population groups. It seems that both aspects of health (SRH and mental health) in the two Arab towns are better than in the Jewish or mixed towns (Table 2).

In a logistic regression of the total population, adjusting for gender, age and education, there was no difference in SRH between Arabs and Jews, however, mental health was worse among Jews after the adjustment (data not presented).

Residents of town A by far reported higher levels of loneliness compared to the other respondents, 38.4% reported feeling lonely often or sometimes, whereas in the other towns, both Arab and Jews, reported between 14.1% and 25.3% feeling lonely often or sometimes. Respondents in town B reported the lowest levels of loneliness (Table 2).

The cognitive measure of SC (trust and reciprocity) was highest in the Arab towns A and B, and lowest in Jewish town D. However, within town C the Arabs had significantly lower levels of cognitive SC compared to Jews. Another measure of SC was the levels of participation in social activities (structural SC), these were higher in the two Arab towns A and B. In town C, Jews reported significantly higher levels of social activities compared to Arabs. Generally, respondents reported spending time with family and friends and going to parties most frequently (Table 2). 

The activities reported varied significantly between the towns, going to a movie was more frequent in towns C and D. Spending time with family, attending sports events, parties and outings were more frequent in town A, and attending prayers in a prayer house was the most frequent activity in town B compared to the rest. This suggests the towns differ in their patterns of social lifestyle.

Logistic and linear regressions with cognitive and structural SC respectively as the dependent variable were run, adjusting for age, gender, education and religiosity (Table 3). Loneliness is associated with cognitive SC only in town C, where those reporting loneliness also reported lower cognitive SC (OR = 0.68, CI = 0.49, 0.94). Being religious is significantly associated with high cognitive SC in the total sample and cities A and D. Gender, age and education are not associated with cognitive SC in the total population and vary in their association by town.

Structural SC (social activities) was significantly inversely associated with loneliness in three of the four towns (towns A, C and D). This association was not significantly different between the towns (*p* > 0.05). Generally, structural SC is more dependent on gender, age and education compared to cognitive SC that is associated only with religiosity in the total population (Table 3).

Table 4 presents the logistic regressions for all the study population and separately for each town with SRH or mental health as the dependant variable and participation in social activities as the measure of structural SC. In these logistic regressions, we adjusted for gender, age, education and religiosity. As expected, when analysing all towns together the more respondents reported being lonely the worse was their self-rated health (SRH) (OR 0.48, CI 0.40, 0.58) and they reported worse mental health (OR 1.94, CI 1.66, 2.27). Structural SC was also significantly associated with SRH and mental health in the total population. We then added the interaction between loneliness and structural SC to test moderation effects (model 2). The interaction between loneliness and structural SC (participation in social activities) was significant for both types of health measures, while the strength of the association between structural SC and SRH or mental health decreased in the total population. To further explore the moderation effect, we analysed the association between loneliness and SRH and mental health of the total population, in two separate logistic regressions, one included respondents reporting low structural SC (range 1–12) and another regression with respondents reporting high structural SC (range 13–24). The association between loneliness and SRH was stronger among people with low structural SC (OR = 0.34, C.I-0.26, 0.46) than among people with high structural SC (OR = 0.58, C.I 0.46, 0.73). Similar results were revealed in relation to mental health. The association between loneliness and mental health was stronger among people with low structural SC (OR = 4.58, C.I-3.31, 6.32) than among people with high structural SC (OR = 1.42, C.I 1.18, 1.71). There was no significant moderation effect with cognitive SC, both for SRH and mental health (data not presented in the table). Adding town to the interaction did not yield a significant interaction (Table 4).

When running the regressions separately for each town with SRH or mental health as the dependant variable, loneliness was associated with SRH and mental health in towns B, C, and D, but not in town A. Structural SC was associated with SRH in all towns. In towns B, C and D, the addition of the interaction decreased the association between structural SC and SRH leaving it not significant. The interaction between loneliness and structural SC did not reach significance when the regressions were run separately for each town (Table 4).

To test the mediation effect of structural SC on the association between loneliness and SRH we calculated the mediation analysis for the total sample and each town separately.

The mediation effect of structural SC was significant for the total population, when including the interaction between town and loneliness. The ACME was significant (the estimate was 0.017 and *p* > 0.001). When running the regressions separately for each town the mediation remained significant for each town: in town A (ACME = 0.012, *p* < 0.0001), town B (ACME = 0.014 *p* = 0.04), town C (ACME = 0.016, *p* = 0.002), town D (ACME = 0.016, *p* < 0.001). Therefore, it seems that structural SC is a mediator in the association between loneliness and SRH. 

Structural SC (participation in social activities) was also a significant mediator in the association between loneliness and mental health in the total population including the interaction between town and loneliness, (ACME = 0.0075, *p* < 0.0001). When running the regressions separately for each town the mediation was significant in town C (ACME = −0.008, *p* < 0.018) and town D (ACME = −0.01, *p* = 0.012) but not in the Arab towns A and B.

Table 5 presents the logistic regressions for all the study population and separately for each town with SRH or mental health as the dependant variable and cognitive SC and loneliness as the independent variables. Again, we adjusted for gender, age, education and religiosity.

Cognitive SC was associated with both SRH and mental health in the total population, where respondents with higher cognitive SC reported better health. Adding the interaction of loneliness and cognitive SC to the model decreases the association, leaving it non-significant. The interaction was not significant both for mental health and SRH, in the total population.

Cognitive SC was associated with SRH in towns C and D (OR = 1.54 CI = 0.99, 2.39 for town B, OR = 1.42 CI = 1.00, 2.01 for town C and OR = 1.61 CI = 1.17, 2.17 for town D), however it was significant (*p* < 0.05) only in town D. Adding the interaction eliminated the significance. The interaction was significant only in town C, therefore there may be a significant moderating effect in town C.

Cognitive SC was significantly associated with mental health in towns A, C and D, however, the direction varied. In Town A, the OR was 1.42 whereas in towns C and D the OR was 0.70 and 0.46 respectively. Adding the interaction eliminated the significance in all three towns. The interaction was significant for the two towns C and D. This suggests that cognitive SC may be a moderator in the association between loneliness and mental health in towns C and D (Table 5).

To test the mediation effect of cognitive SC on the association between loneliness and SRH we used the mediation analysis for the total sample and each town separately. There was no significant mediating effect for cognitive SC in the association between loneliness and SRH in the total sample (ACME = −0.0015, *p* = 0.12) or any of the towns separately. Cognitive SC was also not a significant mediator between loneliness and mental health in the total sample, (ACME = 0.0004 *p* = 0.3) and in the towns separately.

Structural SC may serve as a moderator and a mediator in the association between loneliness and health, both SRH and mental health. This seems not to be true for all towns. Cognitive SC may be a moderator only for mental health and only in two of the towns, however not a mediator.

It seems that the different components of SC vary in the way they mediate or moderate the ill effects of loneliness in the various communities and the two health measures.

## 4. Discussion

This study tries to understand if social capital (SC), both structural and cognitive, can help alleviate some of the ill effects of loneliness on health in certain communities. Studying these associations might support communities and social services in directing their investments to prevent the ill effects of loneliness on health. The model we tested, which presents the association between loneliness and health (SRH and mental health) and the effect of social capital on that association, indicated differences between cities. The direct association between loneliness and Self Reported Health and mental health, which was found in 3 of the 4 examined cities, is consistent with many previous studies [10,39,40].

As for the effect of social capital on this association, we separated the two components of social capital as was suggested by Rodgers and colleagues that each component of social capital represents a very different attribute of the community and may have very different effects on people in general. Putting them together under the umbrella of social capital does not suggest they have the same impact on society [41]. As part of the model, we analyzed the association of each social capital component with SRH and mental health. As in most previous studies, both cognitive and structural social capital were found to be positively associated with self-reported health in the total population and every town [16,17,18,19,20,21]. Less consistent findings were revealed for the association of social capital and mental health, as was reported by De Silva [15]. In 3 out of 4 cities, higher structural and cognitive social capital were associated with better mental health. In a review of cross-sectional and longitudinal studies measuring the association between social capital and common mental disorders [42] a negative association was found with cognitive social capital, but they did not find associations with structural social capital, except for studies among mothers in low-income settings, where participation in civic activities was positively associated with common mental disorders. Such a positive association we found only in town A. It seems that the type of social activities may influence the direction of the association. Croezen and his coauthors found that participation in religious organizations is negatively associated with depressive symptoms while participating in sports or social clubs or political organizations are positively associated with depressive symptoms [43]. In our analysis, we did not differentiate between types of social activities.

Findings suggest that structural social capital serves both as a significant mediator and a moderator in the association between loneliness and health, both SRH and mental health. We found a stronger association between loneliness and SRH and mental health among people with a low level of structural social capital compared to those with high structural social capital. Therefore, it is possible that being in contact with other people via participation in social activities may serve as a possible intervention to dampen or alleviate the ill effects of loneliness on health. Coll-Planas et al. suggest it is an understudied intervention strategy [29]. In a qualitative focus group study, they describe the ways in which a weekly group-based program can alleviate loneliness among older adults by promoting peer support and participation [44]. In our present study, we suggest structural social capital may improve self-reported health in all communities, however, it may improve mental health only in two of the communities, not in the Arab communities. It seems that generalizing from one community to the other is not possible and we suggest that before attempting to utilize a specific intervention it should be studied in that specific community. In other words, the contextual social environment is a major attribute to the way loneliness and social capital may affect health.

Cognitive social capital does not have a mediating effect. A moderation effect was found in relation to self-reported health in one town only and in relation to mental health in two of the towns. In the communities studied in these cross-sectional surveys, trust in social surroundings does not necessarily improve health.

Several interesting differences were found between the towns, for example, in town A, which is the poorest town, the highest rates of loneliness were reported. This is consistent with the findings of the highest risk of loneliness in the least wealthy groups, which were described in the Survey of Health, Ageing and Retirement in Europe (SHARE) [45,46]. Loneliness in town A was negatively associated with structural social capital and the latter was associated with self-reported health, but there was no direct association between loneliness and SRH (controlling for age, gender, education, religiosity and structural social capital), as was found in the other towns. This may indicate that town A may differ from the other towns in its contextual social environment. Poverty may explain the differences to some extent, but of course, there may be other explanatory social factors not measured [47].

The social and cultural differences between the two populations (Arabs and Jews) are large, with the Arabs being a poorer population, and more collective [31,32]. The differences between the communities correspond with national data [48]. However, there were also differences between the two Arab towns in our study.

The moderation effects identified in this study suggest that increasing the social contacts of lonely people, who may have a decreased ability to utilize resources available to help with their health, may affect their health by providing these resources to them. Cognitive social capital was significantly associated with both self-reported health and mental health in 3 out of the 4 towns. In town A, a weak association was detected with self-reported health and an association with mental health in the opposite direction. Similar to the results of town A in this study, in a previous study, trust was not associated with health among Arabs, however, in the Jewish community, it was [25]. The authors suggested that in more affluent communities, such as the Jewish communities, social capital may be associated with health to a higher extent and less so in more traditional communities. It may be that the concept of trust varies between the two communities, the more individualistic community and the more collective community. The lack of association between trust and self-reported health was reported in another study from South Africa [49], where the authors state that the results contradict many studies that do show these associations [50,51,52]. It seems that the impact of social capital on health varies with the setting, population and health measure [53].

Religiosity may also serve as a factor in the social context, as there were differences in religiosity between the towns, therefore it was important to adjust for this measure. Studies have shown that loneliness is more common in people without strong religious beliefs [54,55]. In this study, religiosity was associated with mental health in the total population and in the Arab town A but not in the other towns. Religiosity was associated with self-reported health only in the Jewish town D and not in any of the other towns, again suggesting variations in the social context in the different towns.

Further studies should look at levels of collectivism that could have an impact on these relationships. Dykstra (2009) suggested that contrary to the assumption that people in individualistic societies are more lonely, northern Europeans tend to be less lonely compared to the more familistic southern European countries [56]. This corresponds with our results.

Another aspect of the differences between the communities may be a cultural difference in how the different communities perceive loneliness. The questions used in this survey may vary in their validity and reliability between the communities, not only between Arabs and Jews, as suggested in several previous studies [57,58], but also between the Arab communities themselves, or the Jewish communities. In town A, people may tend to express and complain about feeling alone, more so than in other communities. As people in town A reported higher levels of loneliness, we would expect them to report less social activities, however, higher levels of participation in social activities were reported in town A compared to the other towns. As social activities were associated with lower levels of loneliness in all towns, the more activities within a community the less loneliness we would expect to find. The fact that even though people in town A reported high levels of loneliness and high levels of social activities suggests that in town A social activities do not alleviate loneliness in the same way as in the other towns [59,60], or as mentioned earlier, it may depend on the types of social activities [43].

Both social capital measures were higher in the Arab towns and lower in the mixed and Jewish towns. Other studies in the past suggested otherwise, where social capital measures were lower among Arabs [25]. The only measure of social capital that was higher among Arabs was social contacts, this measure may be a similar measure of social activities, even though they are measured in a different way. As the Arab community is a more collective community where close contacts are frequent, this is expected. However, in the previous study trust was higher in the Jewish community compared to the Arab community, but not so in this study. This may have changed with time and now present a true difference between the communities, or it may also be that town D, as the only Jewish town present in this study, do not represent other Jewish towns. In a study assessing acquiescence bias among Arabs and Jews, we found that Arabs tend to agree with questions more so than Jews [58]. Therefore, the higher rates among Arabs in this study could be due to this bias and not a true measure of their social capital.

In town C, the patterns of association between the variables in this study are similar to the Jews in town D, especially for the Arab community when dividing the sample into the two communities, Arabs and Jews. Living in close proximity in the same town may have an effect on both communities. For example, Arabs in town C gave estimates of their health more similar to the Jewish population than to the Arab population in towns A and B, this may only be the effect of the SES level but may also represent the effect of cohabitation. In addition, social capital was lower in town C compared to towns A and B and more similar to town D. Therefore, it seems that living in a mixed town (town C) reduces social differences between the two ethnic communities.

We may conclude that communities differ in the way social capital serves as a resource within that specific community and depend on the context of a certain community, for example, socioeconomic status and levels of collectivism. What specific attributes of a community are important in this context are not clear yet. In an important paper, Shiell et al. suggested rethinking social capital and social capital interventions aimed at improving health [30]. They suggest that studies measuring social capital and health inadequately take into account the context in which the study takes place. In their opinion, the way forward requires a renewed focus on specific components of social capital and how context affects the interaction between social capital and health. Our study supports these assertions.

A few study limitations should be mentioned. The study is a cross-sectional study that cannot infer causality, therefore follow up studies are needed to ascertain the directionality of the associations between social capital, loneliness and health. The cross-sectional methodology is not the optimal methodology in many cases. However, according to Spector [61], the cross-sectional methodology is appropriate when the association between variables is not clear, the timeframe for the effect is not known and we want to measure the association in four distinct communities. This methodology can help assess preliminary ideas on how we can move forward to alleviate the ill effects of loneliness on health.

The measure of loneliness is a single-item measure and as such is not ideal, however, there is no consensus on how to measure loneliness to date, standardisation is needed [62]. In other studies, certain parts of social capital are incorporated into the loneliness measure, such as frequency of contacts with friends, family and children and participation in social activities [47]. In this study, we assessed perceived loneliness as a single item and refer to levels of contact with other people separately as structural social capital. We do not regard this as a limitation of the study, on the contrary, separating the two aspects of loneliness provides a more in-depth analysis of factors effecting of loneliness and health.

An additional limitation of the study is the possible difference in the validity and reliability of the measures in Arabs and Jews. This has previously been discussed.

A broader look at the context of the communities is needed to further understand the complex dynamics between the studied variables and the possibility of how changing social capital can serve as an intervention to improve health [30].

## 5. Conclusions

This study suggests that increasing structural social capital could possibly compensate for loneliness and alleviate the harm it can do to health. However, we cannot generalize from one community to the other or for the various components of social capital. Therefore, this study reinforces the need for the performance of separate health profiles and the assessment of possible interventions in each community separately.

We may say that structural social capital is both a mediator and a moderator for the total sample with variations between the towns.

## Figures and Tables

**Table 1:** Selected characteristics of the study in each town, mean, standard deviation (SD) or percent and number.

	Town A N = 981	Town B N = 673	Town C N = 1031		Town D N = 1935	P between Towns
			Jews N = 787	Arabs N = 244		
	Mean (SD)	Mean (SD)	Mean (SD)	Mean (SD)	Mean (SD)	
**Age—mean years**	44.4 (15.3)	47.4 (15.2)	52.4 (17.11)	47.0 (15.56)	51.2 (15.3)	<0.001
P §			<0.001			
	%	%	%	%	%	
**Gender**						0.13
Women	51.0	55.3	55.6	53.3	55.2	
Men	49.0	44.7	44.4	46.7	44.8	
P §			0.28			
**Education**						<0.001
Less than 12 years	29.4	31.5	32.8	53.1	17.8	
12–14	49.3	46.7	50.0	37.1	42.3	
15 and above	20.3	18.0	17.2	9.8	39.7	
P §			<0.001			
**Religious**	72.3	91.9	33.0	41.8	11.4	<0.001
Not religious	27.3	8.1	67.0	58.2	88.6	
P §			0.008			

§ *p* value for difference between Jews and Arabs in town C.

**Table 2:** Distribution of Self Rated Health (SRH), mental health, loneliness and social capital variables in each town, mean, standard deviation (SD) or percent and number.

	Town A N = 981	Town B N = 673	Town C N = 1050		Town D N = 1935	P between Towns
			Jews	Arabs		
	%	%	%	%	%	
**Self-rated health-(SRH)**						<0.001
Good (1)	76.7	78.6	73.5	66.1	67.4	
Poor (0)	23.3	21.4	26.5	33.9	32.6	
P §			0.017			
**Mental health**						<0.001
Good (0)	50.1	52.8	42.5	44.1	35.6	
Poor (1)	49.9	47.2	57.5	55.9	64.4	
P §			0.35			
**Loneliness**						<0.001
Never	36.4	78.0	67.2	55.5	48.8	
Infrequently	24.8	8.0	11.2	19.2	30.1	
Sometimes	30.5	9.7	12.6	13.1	18.0	
Often	7.9	4.4	9.0	12.2	3.2	
P §			0.002			
**Cognitive Social Capital**						<0.001
Low (0)	42.0	38.7	41.2	51.0	76.3	
High (1)	58.0	61.3	58.8	49.0	23.7	
P §			0.024			
**Structural Social Capital Mean (** **SD)**	15.8 (2.76)	15.1 (3.06)	11.8 (2.39)	10.2 (2.75)	13.6 (2.32)	<0.001
P §			<0.001			

§ *p* value for difference between Jews and Arabs in town C.

**Table 3:** Variables associated with social capital, logistic regressions presenting odds ratio (OR) and confidence intervals (CI), and linear regression.

Town	Independent Variables	Cognitive Social Capital Logistic Regression		Structural Social Capital Linear Regression	
		OR	CI	Beta adj	*p*
**Town A** N = 954	Gender	**0.76**	0.58, 0.99	**−0.34**	<0.001
	Age	0.10	0.99, 1.01	**−0.14**	<0.001
	Education	1.07	0.86, 1.33	**0.26**	<0.001
	Religiosity	**0.65**	0.47, 0.89	−0.02	0.61
	Loneliness	0.77	0.59, 1.01	**−0.08**	0.004
**Town B** N = 613	Gender	0.90	0.64, 1.27	**−0.24**	<0.001
	Age	0.99	0.98, 1.00	**−0.12**	0.004
	Education	**0.76**	0.59, 0.99	**0.23**	<0.001
	Religiosity	0.65	0.35, 1.19	−0.05	0.25
	Loneliness	0.81	0.50, 1.32	−0.05	0.15
**Town C** N = 1022	Gender	1.26	0.97, 1.65	**−0.18**	<0.001
	Age	1.01	1.00, 1.02	**−0.11**	0.001
	Education	**1.75**	1.42, 2.15	**0.15**	<0.001
	Jews (1) vs. Arabs (2)	0.86	0.63, 1.16	**−0.16**	<0.001
	Religiosity	1.10	0.84, 144	**−0.09**	0.003
	Loneliness	**0.68**	0.49, 0.94	**−0.16**	<0.001
**Town D** N = 1735	Gender	**1.26**	1.01. 1.58	**−0.07**	0.003
	Age	1.00	0.99, 1.01	**−0.19**	<0.001
	Education	0.94	0.79, 1.11	**0.10**	<0.001
	Religiosity	**0.38**	0.28, 0.53	−0.03	0.18
	Loneliness	0.98	0.73, 1.30	**−0.12**	<0.001
**Total population** N = 4329	Gender	1.01	0.89, 1.14	**−0.17**	<0.001
	Age	0.99	0.99, 1.00	**−0.21**	<0.001
	Education	0.91	0.83, 1.00	**0.19**	<0.001
	Religiosity	**0.40**	0.36, 0.46	**−0.23**	<0.001
	Loneliness	0.88	0.76, 1.02	−0.02	0.11

Note: The reference categories of the dichotomized variables are: Gender = male, religiosity = religious, loneliness = not lonely, *p* < 0.05 Bold.

**Table 4:** Variables associated with self-rated health (SRH) and mental health, including structural social capital (participation in social activities). Logistic regressions presenting odds ratios (OR) and confidence intervals (CI).

Town	Independent Variables	SRH				Mental Health			
		Model 1		Model 2		Model 1		Model 2	
		OR	CI	OR	CI	OR	CI	OR	CI
**Total** N = 4279	Gender	0.87	0.74, 1.03	0.88	0.74, 1.04	**1.15**	1.01, 1.30	**1.14**	1.00, 1.29
	Age	**0.93**	0.92, 0.93	**0.93**	0.92, 0.93	**1.02**	1.02, 1.02	**1.02**	1.01, 1.02
	Education	**1.15**	1.02, 1.30	**1.15**	1.02, 1.30	**1.15**	1.04, 1.26	**1.15**	1.04, 1.26
	Religiosity	1.13	0.94, 1.35	1.13	0.94, 1.35	**1.17**	1.03, 1.35	**1.18**	1.03, 1.35
	Loneliness	**0.48**	0.40, 0.58	**0.26**	0.06, 0.40	**1.94**	1.66, 2.27	**10.14**	4.53, 22.7
	Structural Social capital	**1.14**	1.10, 1.18	1.02	0.93, 1.12	**0.92**	0.90, 0.94	1.06	0.99, 1.14
	Loneliness * Structural Social capital	-	-	**1.09**	1.02, 1.17			**0.89**	0.84, 0.94
**Town A** N = 941	Gender	0.95	0.65, 1.40	0.94	0.64, 1.37	1.07	0.80, 1.41	1.07	0.81, 1.42
	Age	**0.95**	0.94, 0.97	**0.95**	0.94, 0.97	1.00	0.99, 1.03	1.00	0.99, 1.01
	Education	1.31	0.80, 2.07	1.31	0.96, 1.78	0.88	0.70, 1.10	0.88	0.70, 1.10
	Religiosity	1.29	0.81, 2.10	1.31	0.81, 2.10	**0.60**	0.44, 0.82	**0.60**	0.44, 0.82
	Loneliness	0.85	0.59, 1.21	4.19	0.54, 32.3	0.79	0.60, 1.04	0.43	0.09, 2.09
	Structural Social capital	**1.18**	1.10, 1.27	**1.37**	1.11, 1.68	0.98	0.93, 1.04	0.93	0.81, 1.08
	Loneliness * Structural Social capital	-	-	0.90	0.79, 1.03			1.04	0,94, 1.15
**Town B** N =611	Gender	1.00	0.63, 1.60	1.00	0.63, 1.61	1.15	0.82, 1.62	1.15	0.82, 1.62
	Age	**0.95**	0.94, 0.97	**0.95**	0.94, 0.97	1.00	0.99, 1.02	1.00	0.99, 1.02
	Education	**1.56**	1.07, 2.28	**1.57**	1.08, 2.30	1.06	0.81, 1.38	1.06	0.81, 1.38
	Religiosity	0.80	0.33, 1.94	0.81	0.33, 1.97	1.56	0.83, 2.91	1.56	0.83, 2.91
	Loneliness	**0.47**	0.26, 0.83	**0.23**	0.01, 5.44	**2.89**	1.73, 4.82	1.70	0.12, 23.4
	Structural Social capital	**1.19**	1.09, 1.30	**1.12**	0.86, 1.47	0.97	0.92, 1.03	0.94	0.76, 1.15
	Loneliness * Structural Social capital	-	-	1.05	0.84, 1.31			1.04	0.87, 1.24
**Town C** N= 937	Gender	0.83	0.57, 1.21	0.83	0.57, 1.12	1.30	0.98, 1.72	1.30	0.98, 1.72
	Age	**0.94**	0.93, 0.95	**0.94**	0.92, 0.95	1.01	1.00, 1.02	1.01	1.00, 1.02
	Education	**1.49**	1.14, 1.94	**1.49**	1.14, 1.94	1.00	0.81, 1.25	1.00	0.81, 1.25
	Religiosity	1.25	0.86, 1.27	1.25	0.86, 1.82	1.16	0.86, 1.55	1.16	0.86, 1.55
	Loneliness	**0.26**	0.17, 0.39	**0.07**	0.01, 0.66	**9.50**	5.64, 16.0	14.1	0.94, 213
	Jews (1) Vs. Arabs (2)	**0.63**	0.40, 0.99	**0.58**	0.38, 0.89	0.89	0.62, 1.28	0.89	0.62, 1.28
	Structural Social capital	**1.10**	1.01, 1.20	0.95	0.74, 1.23	1.06	0.99, 1.13	1.10	0.84, 1.44
	Loneliness * Structural Social capital	-	-	1.12	0.92, 1.36			0.96	0.76, 1.23
**Town D** N = 1785	Gender	0.87	0.67, 1.14	0.86	0.66, 1.13	1.12	0.90, 1.38	1.11	0.90, 1.38
	Age	**0.89**	0.88, 0.90	**0.89**	0.88, 0.90	**1.03**	1.03, 1.04	**1.03**	1.03, 1.04
	Education	1.09	0.90, 1.33	1.09	0.90, 1.33	1.17	1.00, 1.38	1.17	0.99, 1.38
	Religiosity	**1.79**	1.15, 2.77	**1.81**	1.17, 2.80	1.02	0.72, 1.45	1.02	0.72, 1.45
	Loneliness	**0.59**	0.42, 0.82	**0.11**	0.02, 0.85	**2.64**	1.91, 3.64	1.24	0.20 7.84
	Structural Social capital	**1.27**	1.27, 1.36	1.09	0.90, 1.32	**0.77**	0.73, 0.82	**0.72**	0.62, 0.85
	Loneliness * Structural Social capital	-	-	1.13	0.98, 1.32			1.06	0.93, 1.20

Note: The reference categories of the dichotomized variables are: Gender = male, religiosity = religious, loneliness = not lonely, * *p* < 0.05 bold.

**Table 5:** Variables associated with self-rated health (SRH) and mental health, including cognitive social capital. Logistic regressions presenting odds ratio (OR) and confidence intervals (CI).

Town	Independent Variables	SRH				Mental Health			
		Model 1		Model 2		Model 1		Model 2	
		OR	CI	OR	CI	OR	CI	OR	CI
**Total** N = 4279	Gender (Men-1, Women-2)	**0.76**	0.65, 0.89	**0.76**	0.65, 0.90	**1.12**	1.01, 1.41	**1.24**	1.09, 1.41
	Age	**0.92**	0.92, 0.93	**0.92**	0.92, 0.93	**1.02**	1.02, 1.03	**1.02**	1.02, 1.03
	Education	**1.30**	1.16, 1.46	**1.30**	1.15, 1.46	1.07	0.97, 1.17	1.07	0.97, 1.18
	Religiosity	1.03	0.86, 1.22	1.03	0.87, 1.23	**1.22**	1.07, 1.39	**1.22**	1.06, 1.39
	Loneliness	**0.44**	0.37, 0.53	**0.42**	0.34, 0.51	**1.98**	1.69, 2.32	**2.08**	1.74, 2.49
	Cognitive social capital	**1.57**	1.33, 1.86	1.29	0.94, 1.78	**0.72**	0.63, 0.82	0.82	0.64, 1.04
	Loneliness * Cognitive social capital	-		1.11	0.96, 1.29	-		0.93	0.83, 1.05
**Town A** N = 941	Gender (Men-1, Women-2)	**0.70**	0.49, 1.00	**0.69**	0.49, 0.97	1.14	0.87, 1.48	1.14	0.88, 1.49
	Age	**0.95**	0.94, 0.97	**0.95**	0.94, 0.97	1.00	0.99, 1.01	1.00	0.99, 1.01
	Education	**1.53**	1.14, 2.06	**1.56**	1.16, 2.10	0.86	0.69, 1.07	0.84	0.67, 1.05
	Religiosity	1.28	0.80, 2.06	1.29	0.81, 2.07	**0.62**	0.45, 0.85	**0.62**	0.45, 0.84
	Loneliness	0.79	0.56, 1.12	0.94	0.62, 1.43	0.81	0.61, 1.06	**0.71**	0.51, 0.98
	Cognitive social capital	1.12	0.79, 1.58	1.77	0.97, 3.62	**1.43**	1.10, 1.87	1.00	0.58, 1.71
	Loneliness * Cognitive social capital	-		0.82	0.64, 1.07	-		1.17	0,95, 1.44
**Town B** N = 611	Gender	0.77	0.45, 1.20	0.77	0.49, 1.20	1.20	0.86, 1.67	1.20	0.86, 1.67
	Age	**0.95**	0.93, 0.97	**0.95**	0.93, 0.97	1.00	0.99, 1.07	1.00	0.99, 1.02
	Education	**1.89**	1.31, 2.73	**1.89**	1.31, 2.73	1.03	0.78, 1.33	1.03	0.81, 1.33
	Religiosity	0.76	0.32, 1.83	0.80	0.33, 1.94	1.54	0.82, 2.88	1.51	0.81, 2.84
	Loneliness	**0.45**	0.26, 0.80	**0.38**	0.20, 0.72	**2.86**	1.71, 4.78	**2.89**	1.79, 5.73
	Cognitive social capital	1.54	0.99, 2.39	1.05	0.47, 2.34	0.82	0.58, 1.14	1.02	0.55, 1.87
	Loneliness * Cognitive social capital	-		1.28	0.83, 1.98	-		0.87	0.62, 1.22
**Town C** N = 937	Gender	**0.69**	0.48, 0.99	**0.69**	0.48, 0.99	1.30	0.98, 1.69	1.29	0.98, 1.69
	Age	**0.93**	0.92, 0.94	**0.93**	0.92, 0.94	1.01	1.00, 1.02	1.01	1.00, 1.02
	Education	**1.58**	1.22, 2.05	**1.55**	1.19, 2.01	1.05	0.84, 1.29	1.07	0.86, 1.32
	Religiosity	1.26	0.89, 1.80	1.29	0.91, 1.85	1.11	0.84, 1.47	1.09	0.82, 1.45
	Loneliness	**0.23**	0.15, 0.33	**0.17**	0.11, 0.27	**9.00**	5.60, 14.4	**12.2**	7.01, 21.3
	Jews (1) Vs. Arabs (2)	**0.49**	0.33, 0.74	**0.48**	0.32, 0.73	0.91	0,65, 1.27	0.92	0.66, 1.28
	Cognitive social capital	1.42	1.00, 2.01	0.74	0.39, 1.38	**0.70**	0.54, 0.93	1.19	0.72, 1.95
	Loneliness * Cognitive social capital	-		**1.49**	1.08, 2.04	-		**0.70**	0.52, 0.93
**Town D** N = 1785	Gender	0.83	0.64, 1.09	0.83	0.63, 1.08	1.21	0.98, 1.50	1.21	0.98, 1.50
	Age	**0.88**	0.87, 0.89	**0.88**	0.87, 0.89	**1.04**	1.03, 1.05	**1.04**	1.03, 1.05
	Education	1.17	0.96, 1.42	1.17	0.96, 1.42	1.07	0.91, 1.26	1.07	0.91, 1.26
	Religiosity	**1.68**	1.08, 2.62	**1.66**	1.07, 2.58	0.91	0.64, 1.31	0.93	0.65, 1.34
	Loneliness	**0.47**	0.34, 0.65	**0.42**	0.29, 0.59	**2.88**	2.08, 3.97	**3.37**	2.35, 4.82
	Cognitive social capital	**1.61**	1.17, 2.17	0.91	0.47, 1.78	**0.46**	0.36, 0.59	0.78	0.47, 1.30
	Loneliness * Cognitive social capital	-		1.40	0.99, 1.98	-		**0.73**	0.56, 0.96

Note: The reference categories of the dichotomized variables are: Gender = male, religiosity = religious, loneliness = not lonely, * *p* < 0.05 Bold.

## Data Availability

Data available on request.
